# Illicit Substance Use and Harm in Young Adulthood: the Role of Substance Use in Close Relationships and Individual Social Skills

**DOI:** 10.1007/s11469-023-01181-0

**Published:** 2023-10-27

**Authors:** C. J. Greenwood, P. Letcher, J. A. Macdonald, D. M. Hutchinson, G. J. Youssef, J. W. Toumbourou, E. Spry, A. Sanson, J. Cleary, C. A. Olsson

**Affiliations:** 1 Centre for Social and Early Emotional Development, School of Psychology, Faculty of Health, Deakin University, Geelong, Australia; 2 Centre for Adolescent Health, Murdoch Children’s Research Institute, Melbourne, Australia; 3 Department of Paediatrics, Royal Children’s Hospital, University of Melbourne, Melbourne, Australia; 4 National Drug and Alcohol Research Centre, Faculty of Medicine, University of New South Wales, Sydney, Australia

**Keywords:** Illicit Substance Use, Substance Use Harm, Peer and Partner Use, Social Skills, Young Adulthood, Interaction

## Abstract

To examine illicit substance use across young adulthood and explore the extent to which social skills moderate the relationship between use and harm. 1,404 (761 women) from the Australian Temperament Project (est. 1983) participated across young adulthood (age 19–20, 23–24, and 27–28 years). Measures included self-reported illicit substance use/harm and social skills (i.e., assertion, empathy, responsibility, and self-control). The number and type of illicit substances used changed across young adulthood. Greater illicit substance use was associated with peer (OR = 4.96) and partner use (OR = 3.60). Moderation analyses suggested the risk relationship between the number of illicit substances used and harm was lower in those with high levels of assertion/self-control (OR_assertion_ = 2.34, OR_self-control_ = 2.60) compared to low levels (OR_assertion_ = 4.43, OR_self-control_ = 3.72). Evidence based programmes designed to strengthen individual social skills for young adults may play a role in protecting against the adverse effects of illicit substance use.

**Supplementary Information:**

The online version contains supplementary material available at 10.1007/s11469-023-01181-0.

Substance use disorders remain a substantial contributor to the burden of disease in Australia (Australian Institute of Health & Welfare, 2022). In recent years, rates of illicit substance (including cannabis) use have increased slightly, with Australian national estimates of past-year use indicating that use was highest during the emerging and young adult periods (age 20–29 years; 34% males; 23% females) (Australian Institute of Health & Welfare, 2020). Further, recent estimates suggest that amongst people who use substances, there was an increase in poly-substance use (i.e., using multiple drugs at the same time); a trend which substantially increases risk of harm (e.g., addiction, overdose, and other medical problems) (Australian Institute of Health & Welfare, 2020). For these reasons, preventing use and minimising associated harm remain a key priority (Commonwealth of Australia, 2017).

Identification of factors that reduce harms experienced by people who use substances can inform harm minimization efforts aimed at limiting the negative effects of substance use on the individual, as well as their families and peers. Relatedly, the social development model has been an influential framework for several decades for understanding both risk and protection within substance use pathways and has suggested the importance of relational factors (Cambron et al., 2019; Catalano & Hawkins, 1996). The framework posits that substance use behaviours, amongst other deviant behaviours, are learnt and maintained through a sequence of socialisation processes. The initial opportunities for involvement with others develop through repeated interactions and progress into strong social bonds. If these socialisation processes occur amongst individuals using substances, these behaviours are reinforced. Previous work has found that both higher levels of substance use and poly-substance use are associated with greater use of substances in close social relationships (Bahr et al., 2005; Fergusson et al., 2008; Fleming et al., 2010; Tomczyk et al., 2016). Substance use behaviours in the context of close social relationships thus represent key risks for individual patterns of use and harm.

It is evident, however, that not all those who use illicit substances experience similar levels of harm. This may be explained partly by the frequency, amount, and number of substances used. However, even in those with similar patterns of substance use, harm may still be experienced differently (Greenwood et al., 2021; Rehm et al., 2004). One approach to understanding this variation is from a resilience perspective, whereby resilience is an interactive phenomenon for which certain factors buffer (i.e., moderate) risk relationships (Rutter, 2013). In line with this, the social development model further suggests that compliance or resistance to peer socialisation pressures may be moderated by a range of social skills (Catalano & Hawkins, 1996). Social skills including assertion, empathy, responsibility, and self-control (Gresham & Elliott, 1990; Smart & Sanson, 2003) are broadly considered to protect young people despite exposure to risk (Arthur et al., 2002) and have been implicated in reduced risk of substance use disorders (Ham & Garcia, 2010). Prior evidence has suggested that most young adults saw themselves as commonly possessing these skills (Smart & Sanson, 2003). Developing the necessary competencies in these domains is critical to an individual’s ability to engage effectively in close relationships, and in turn, to an individuals’ selection of appropriate responses and behaviours.

However, empirical evidence for the extent to which social skills may protect against (moderate) harms associated with illicit substance use is relatively limited. Self-control, for example, may buffer harmful outcomes associated with substance use, although studies have primarily focused on alcohol, tobacco, and cannabis use rather than other illicit substance types (Simons et al., 2009; Wills et al., 2002, 2011). This may happen both behaviourally (e.g., through forethought and problem-solving capabilities) and/or emotionally (e.g., through the ability to manage states of arousal) (Wills et al., 2011). Further examinations of a wider range of social skills are needed, particularly in the context of illicit substance use and harms.

Here, we aim to use unique developmental data on young adulthood, from one of Australia’s longest running study of social and emotional development to examine: (1) the patterns of illicit substance use across young adulthood, the period when illicit substance use peaks; (2) associations between illicit substance use and use in the context of close relationships (i.e., with peers and partners) and with social skills across young adulthood; and (3) the extent to which social skills may moderate (buffer) associations between illicit substance use and harm, after accounting for peer/partner use.

## Methods

### Participants

Participants were from the Australian Temperament Project (ATP), a 16-wave longitudinal study tracking the psychosocial development of young people from infancy to adulthood. In 1983, 67 local government areas (LGAs) in the state of Victoria were randomly selected based on Australian Bureau of Statistics, to provide a representative community sample (Sanson et al., 1985). Parents of every 4–8-month-old infant who visited one of the selected centres between 22nd of April and 6th of May (1983) were invited to participate. Approximately 3,000 questionnaires were administered and following exclusions (e.g., child outside the appropriate age range, missing data, and questionnaire not returned), the baseline sample consisted of 2,443 infants aged 4–8 months (81% of 3,000). Following cohort recruitment testing procedures in 1984 and 1985, a representative subset of 2,023 families continued to complete mail surveys approximately every 2 years until children were 19–20 years of age (13 waves, 1983–2002), and every 4 years thereafter (3 waves, 2006–2014; from 2010, participants could also opt to complete the survey online) (Vassallo & Sanson, 2013). Parents have participated in surveys since 1983 (baseline) and participants since 1994 (11–12 years).

The study is based on an open cohort design that allowed participants to participate or not on any given wave. Non-respondents were followed up with a second mailout of the survey and phone call reminders. By age 27–28 years, 1,701 participants remained active in the study (84% of the longitudinal cohort). Attrition analyses have suggested minimal bias with only marginally higher loss to follow up in participants with parents who were non-Australian born and had lower education levels (Olsson et al., 2022). Additional examinations revealed that key sociodemographic baseline characteristics were prevalent at similar proportions in each of the young adult waves. Data collection waves were variously approved by Human Research Ethics Committees at the University of Melbourne, the Australian Institute of Family Studies, and/or the Royal Children’s Hospital, Melbourne.

To be included in the current study, at least one wave of relevant data during young adulthood (2002–2012) was required (3 waves; ages 19–20, 23–24, and 27–28 years). The resulting sample size was 1,404 (761 women and 643 men), for which 743 (53%) provided relevant data across all 3 young adult waves (*n* = 308 [22%] 2 waves, *n* = 353 [25%] 1 wave).

### Measures

#### Illicit Substance Use

Illicit substance use was self-reported at each young adult wave (ages 19–20, 23–24, and 27–28 years). Participants endorsed the number of days in the last month that they had used cannabis, hallucinogens (e.g., LSD and magic mushrooms), ecstasy, amphetamines, heroin, cocaine, and sleeping tablets/tranquilisers (without a prescription). At age 23–24 and 27–28 years, amphetamine use was incorporated into the assessment of methamphetamine use. Given the low prevalence of illicit substance use, we derived two variables from these data at each wave for analytic use. The first was a categorical indicator of no use versus any use. The second variable was the sum of the number of illicit substances used. We further developed separate indicators that both included and excluded cannabis use, given the notable differences in prevalence and acceptability of cannabis versus other illicit substance use in Australia (Australian Institute of Health & Welfare, 2020).

#### Illicit Substance Use Harm

Illicit substance use harm was self-reported at each young adult wave (ages 19–20, 23–24, and 27–28 years). Illicit substance use harm was assessed based on the frequency of experiencing harms in the last year separately for any cannabis (5 harms) and non-cannabis illicit substance (6 harms) use. Harms for cannabis use (Cronbach’s α_19–20_ = 0.84, α_23–24_ = 0.94, α_27–28_ = 0.86) included: ‘couldn’t get through the week without it’, ‘it was having a bad effect on your life’, ‘unable to stop using it’, ‘irritable or depressed when unavailable’, and ‘trouble with police’. Harms for non-cannabis illicit substance use (Cronbach’s α_19–20_ = 0.77, α_23–24_ = 0.78, α_27–28_ = 0.84) included: ‘couldn’t get through the week without it’, ‘unable to stop using when you want to’, ‘irritable or depressed when unavailable’, ‘blackouts or flashbacks’, ‘trouble at home, work, or school’, and ‘trouble with police’. Response options at 19–20 years were ‘never’, ‘once or twice’, and ‘more often’ and at 23–24 and 27–28 years were ‘never’, ‘less than monthly’, ‘monthly’, ‘weekly’, and ‘daily or almost daily’. For each wave, mean scores were derived, and then binary variables were defined measuring the endorsement of no harm vs. any harm. Two indicators were used in the current study: any illicit harm (combined cannabis and non-cannabis harm) and any non-cannabis illicit harm.

#### Substance Use in Close Relationships (Peer and Partner)

To indicate the extent to which a young person was likely to be engaged in a social context involving substance use, we further examined peer and partner illicit substance use. These were assessed with single items (peer: ‘How many of your close friends would you say: use marijuana or other drugs’; partner: ‘To what extent would you say your partner/boyfriend/girlfriend: uses marijuana or other drugs’). Responses were coded to indicate any peer use (‘none’ vs. ‘a few’ to ‘most’) and any partner use (‘not true’ vs. ‘somewhat true’ to ‘definitely true’). Participants without a partner were scored as having no partner use.

#### Social Skills

Social skills were assessed at each young adulthood wave via scales developed by Smart and Sanson (2003), based on Gresham and Elliott’s (1990) model of child and adolescent social competence. The four facets assessed were assertion (5 items, e.g., “I initiate conversations in groups”; Cronbach’s α_19–20_ = 0.74, α_23–24_ = 0.75, α_27–28_ = 0.76), responsibility (4 items, e.g., “I fulfil my obligations”; Cronbach’s α_19–20_ = 0.72, α_23–24_ = 0.71, α_27–28_ = 0.72), empathy (5 items, e.g., I try to be a kind and caring person”; Cronbach’s α_19–20_ = 0.79, α_23–24_ = 0.78, α_27–28_ = 0.80), and self-control (3 items, e.g., “I negotiate and compromise with people when we have disagreements”; Cronbach’s α_19–20_ = 0.60, α_23–24_ = 0.59, α_27–28_ = 0.62). For a further description of the items in each scale, see Smart and Sanson (2003). Scores ranged from 1–5, with higher scores indicating greater levels of the respective social skill. Pearson correlations between constructs ranged between 0.32 and 0.46 at 19–20 years, 0.29 and 0.44 at 23–24 years, and 0.29 and 0.44 at 27–28 years.

#### Potential Confounding Factors

A range of potential confounders measured prior to or at the time of the exposure were assessed in order to account for other factors which may explain the associations of interest. These included parent family background characteristics of country of birth (either parent not born in Australia), low parental education (≤ secondary school completion), and separation/divorce. Also included were participant sex (male vs. female), elevated levels of mental health symptoms (depression or anxiety) across adolescence (13–18 years; combined across the Short Mood and Feelings Questionnaire (cut-off ≥ 11) (Angold et al., 1995; Turner et al., 2014), an adapted version of the Revised Behaviour Problem Checklist Short Form (cut-off > 1) (Quay & Peterson, 1987), a short form of the Revised Children’s Manifest Anxiety Scale (cut-off > 1) (Reynolds & Richmond, 1997)), antisocial behaviour across adolescence (13–18 years; two of the following behaviours occurring at least once or one behaviour more frequently: physical fights, damaged something, stolen something, driven a car without permission, been suspended/expelled from school, done graffiti, carried a weapon, and run away from home), and frequent (at least weekly) adolescent substance use (13–18 years; alcohol, tobacco, or cannabis use). We additionally adjust for relationship status (0 = not in a relationship, 1 = in a relationship) at each young adult wave.

### Statistical Analysis

All analyses were conducted in Stata 17 (StataCorp, 2021). To examine the research questions, a series of logistic regression models were estimated using generalised estimating equations (GEEs) with an exchangeable working correlation structure, which assumes a constant correlation between any pair of measurements within the same participant (i.e., repeated measures). First, associations between any illicit substance use and indicators of both social skills and peer/partner substance use were estimated. Second, the association between any illicit harm and the number of illicit substances used was estimated. Associations were estimated in two adjustment steps: (1) including measurement time point only and (2) also including social skills, peer/partner substance use, and potential confounding factors. Third, the potential moderating influence of social skills on associations between substance related harm and the number of illicit substances used was examined by additionally including an interaction term between the number of illicit substances and each social skill indicator accounting for the full set of adjustments (social skills, peer/partner substance use, and potential confounding factors). All associations were also examined within each young adult period by including an interaction between relevant predictors and measurement time point. Primary models used illicit substance use and harm indicators including cannabis use with models excluding cannabis presented in the supplementary material. Social skill variables were standardised (*z*-score) to assist with interpretation.

Missing data within the analysis sample ranged from 0 to 30%. Multiple imputation was used to handle missing data in the inferential analyses. Fifty complete datasets were imputed, based on a multivariate normal model (Lee & Carlin, 2010). Binary variables were imputed as continuous variables and then back transformed with adaptive rounding following imputation (Bernaards et al., 2007). This approach to imputation has been shown to perform similarly to imputation models using chained equations (Lee & Carlin, 2010). The interaction terms between illicit substance use and each social skill were also included. Separate imputation models were conducted for variables including and excluding cannabis. Estimates were obtained by pooling results across the 50 imputed datasets using Rubin’s rules (Rubin, 1987).

## Results

### Descriptives

Descriptive statistics for the full sample are presented in Table 1 (correlation matrix between key study variables presented in the supplementary material Table S1). The prevalence of any illicit substance use (in the last month) varied between 21 and 27% across young adulthood. Non-cannabis illicit substance use varied between 9 and 14% prevalence. Whilst illicit substance use was most prevalent at age 27–28 years, non-cannabis illicit substance use was more prevalent at age 23–24 years. During each young adult age, the most used substance was consistently cannabis. However, the most prevalent non-cannabis illicit substance varied by age; the most prevalent were hallucinogens at age 19–20 years (6%), ecstasy at age 23–24 years (9%), and amphetamines at age 27–28 years (5%). Close to half of all participants reported that they had peers or a partner who used cannabis or other drugs, with higher percentages reported for peer use (44–57%) than partner use (11–12%). Notably, rates of peer use were lower at age 27–28 years in comparison to prior ages (*p* < 0.001 for both age 19–20 and 23–24 years), whilst there were no differences in partner use (*p* > 0.05). Social skill scores were similar across each young adult measurement time point.

**Table 1:** Descriptive statistics for illicit substance use in the full sample (*N* = 1,404)

	19–20 years				23–24 years				27–28 years			
	*n*	%	95% CI	% missing	*n*	%	95% CI	% missing	*n*	%	95% CI	% missing
Illicit substance use	286	25%	(22, 27)	18%	211	21%	(19, 24)	29%	269	27%	(24, 29)	28%
Non-cannabis illicit substance use	101	9%	(7, 11)	18%	141	14%	(12, 16)	29%	104	10%	(9, 12)	28%
Cannabis	242	21%	(19, 23)	18%	136	14%	(12, 16)	29%	226	22%	(20, 25)	28%
Hallucinogens	66	6%	(5, 7)	19%	13	1%	(1, 2)	30%	11	1%	(1, 2)	28%
Ecstasy	1	0%	(0, 1)	25%	91	9%	(8, 11)	29%	39	4%	(3, 5)	28%
Amphetamines	7	1%	(0, 1)	24%	78	8%	(6, 10)	29%	46	5%	(3, 6)	29%
Heroin	15	1%	(1, 2)	19%	1	0%	(0, 1)	29%	1	0%	(0, 1)	28%
Cocaine	6	1%	(0, 1)	19%	44	4%	(3, 6)	29%	35	4%	(3, 5)	29%
Sleep tablets/tranquilisers	24	2%	(1, 3)	20%	22	2%	(1, 3)	29%	33	3%	(2, 5)	28%
Social use												
Any	677	59%	(56, 61)	18%	540	54%	(51, 57)	29%	453	45%	(42, 48)	29%
Peer use	654	57%	(54, 60)	19%	526	54%	(51, 57)	30%	430	44%	(41, 47)	30%
Partner use	136	12%	(10, 14)	21%	114	12%	(10, 14)	32%	100	11%	(9, 13)	33%
	*M*	SD	95% CI	% missing	*M*	SD	95% CI	% missing	*M*	SD	95% CI	% missing
Social skills												
Empathy	4.1	0.6	(4.1, 4.1)	18%	4.1	0.5	(4.1, 4.2)	29%	4.1	0.5	(4.1, 4.1)	26%
Assertion	3.7	0.6	(3.6, 3.7)	18%	3.7	0.6	(3.7, 3.7)	29%	3.6	0.6	(3.6, 3.6)	26%
Responsibility	4.1	0.5	(4.1, 4.1)	18%	4.3	0.5	(4.3, 4.3)	29%	4.3	0.5	(4.3, 4.3)	26%
Self-control	3.6	0.6	(3.6, 3.6)	19%	3.7	0.5	(3.7, 3.8)	29%	3.8	0.6	(3.7, 3.8)	26%

% missing calculated using the full sample for all variables

Patterns of illicit substance use amongst people who reported use are presented in Table 2. Most people who used substances used only one type (range 52 to 79%), with the large majority being exclusive cannabis use, given that when excluding cannabis most people used no substances (range 33 to 64%). At age 23–24 years, there was the greatest proportion of people using more than one type of substance; however, at age 27–28, there was the greatest spread of number of substances use. Harms associated with illicit substance use were reported by 15 to 46% of participants who used substances, with the highest prevalence of harm occurring at age 23–24 years. A similar pattern was observed when excluding cannabis specific harms, although with a lesser prevalence. Most people who used substances also reported that they had peers or a partner using illicit substances (range 83 to 93%).

**Table 2:** Descriptive statistics for illicit substance use amongst people who use substances

	19–20 years			23–24 years			27–28 years		
	*n*	%	95% CI	*n*	%	95% CI	*n*	%	95% CI
# of substances									
1	225	79%	(74, 83)	110	52%	(45, 59)	195	72%	(67, 78)
2	49	17%	(13, 22)	45	21%	(16, 27)	43	16%	(12, 21)
3	10	3%	(2, 6)	41	19%	(15, 25)	18	7%	(4, 10)
4	2	1%	(0, 3)	13	6%	(4, 10)	10	4%	(2, 7)
5	-			2	1%	(0, 4)	2	1%	(0, 3)
6	-			-			1	0%	(0, 3)
# of non-cannabis substances									
0	181	64%	(58, 70)	69	33%	(27, 40)	165	61%	(55, 67)
1	87	31%	(26, 37)	66	31%	(25, 38)	66	25%	(20, 30)
2	10	4%	(2, 6)	46	22%	(17, 28)	21	8%	(5, 12)
3	4	1%	(1, 4)	25	12%	(8, 17)	12	4%	(3, 8)
4	-			4	2%	(1, 5)	4	1%	(1, 4)
5	-			-			1	0%	(0, 3)
Harm									
Any harm	44	15%	(12, 20)	98	46%	(40, 53)	73	27%	(22, 33)
Any harm (exc. cannabis)	13	5%	(3, 8)	50	24%	(19, 30)	29	11%	(8, 15)
Social use									
Any	267	93%	(90, 96)	197	93%	(89, 96)	222	83%	(78, 87)
Peer use	265	93%	(90, 96)	195	93%	(88, 96)	215	81%	(76, 85)
Partner use	77	28%	(23, 33)	69	35%	(28, 42)	63	26%	(21, 32)

% missing calculated using the full sample for all variables; people who use substances includes both cannabis and non-cannabis illicit substance users

### Associations Between Illicit Substance Use and Social Factors 

Associations between any illicit substance use and social factors (i.e., social skills and peer/partner substance use) are presented in Table 3. For social skills, greater responsibility was associated with a reduction in the odds of any illicit substance use (OR = 0.79). Comparatively, greater assertion was associated with an increase in the odds of any illicit substance use (OR = 1.25). Both peer and partner use were associated with an increase in the odds of any illicit substance use (OR_peer_ = 4.96; OR_partner_ = 3.60). Analyses exploring associations excluding cannabis use and across each wave are presented in Table S2 and S3, in which the main pattern of findings was largely unchanged.

**Table 3:** Associations between any illicit substance use and both social skills and social use indicators (*N* = 1,404)

	Model 1		Model 2	
	OR	95% CI	OR	95% CI
Social skills				
Empathy (*z*-score)	0.99	(0.90, 1.08)	0.98	(0.87, 1.11)
Assertion (*z*-score)	1.15	(1.06, 1.26)	1.25	(1.11, 1.39)
Responsibility (*z*-score)	0.79	(0.73, 0.86)	0.79	(0.71, 0.87)
Self-control (*z*-score)	1.02	(0.94, 1.11)	1.05	(0.94, 1.17)
Social use				
Peer use	5.52	(4.40, 6.92)	4.96	(3.92, 6.29)
Partner use	3.08	(2.44, 3.89)	3.60	(2.73, 4.76)

Model 1 = adjusted for measurement time point only; Model 2 = additionally adjusted for social skills, peer/partner substance use, and potential confounding factors; all associations did not differ by participant sex (*p* > 0.05)

### Social Skills Moderating the Association Between Illicit Substance Use and Harm 

**Fig. 1:**
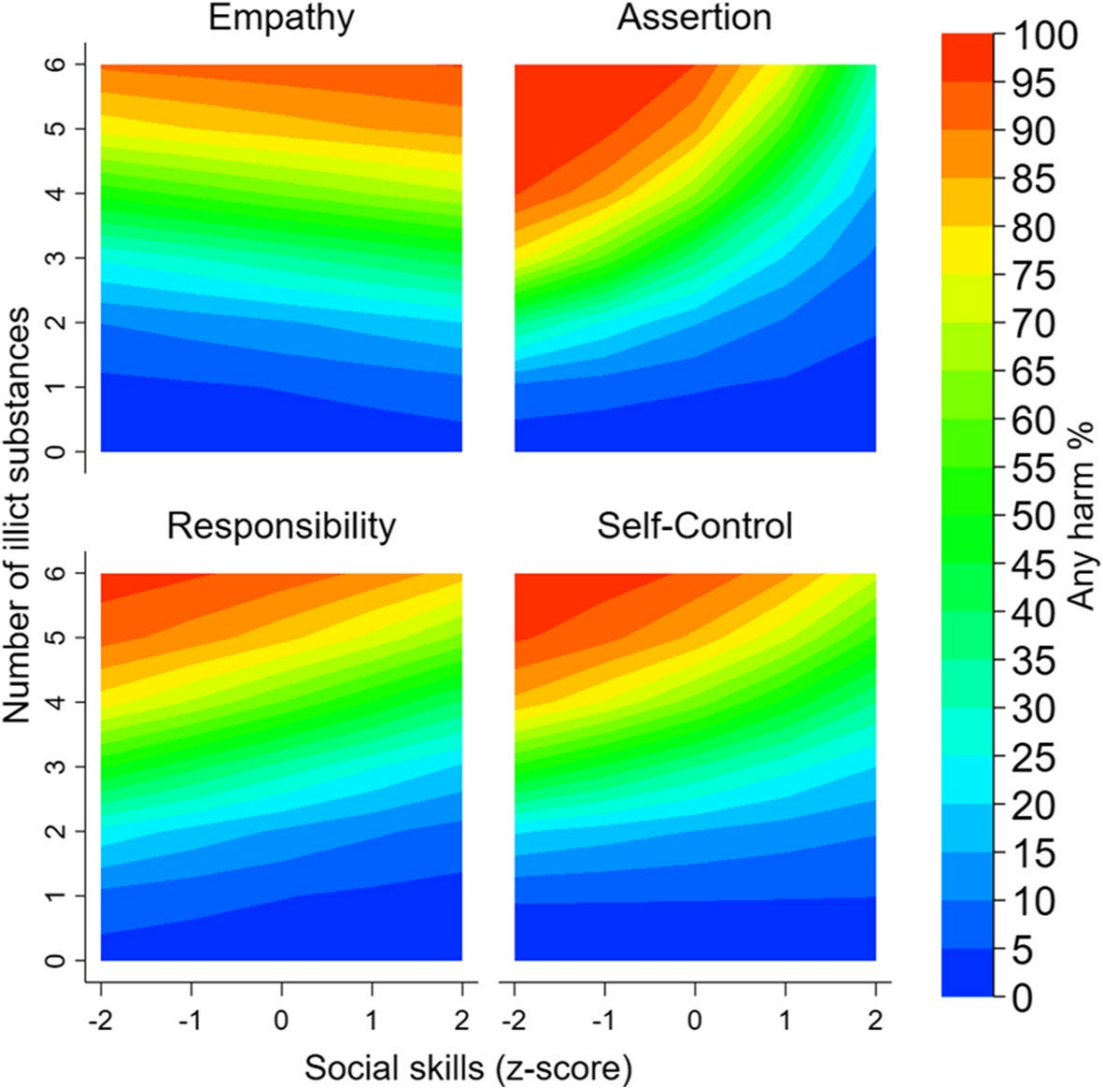
Percentage of participants experiencing harm in the interactive effect of the number of illicit substances and social skills. Note: Number of potential illicit substances including cannabis ranges from 0 to 6

Associations between the number of illicit substances used and harm (main effect associations are presented in Table S4) as moderated by each social skill are shown in Fig. 1. There was evidence to suggest that the association between the number of illicit substances used and the experience of harm was moderated by assertion (*p* < 0.001). The association between the number of illicit substances used and harm was stronger when assertion was low (1SD below the mean; OR = 4.43, 95% CI = 3.31, 5.92), in comparison to when assertion was high (1SD above the mean; OR = 2.34, 95% CI = 1.87, 2.93). Additionally, the association between the number of illicit substances used and harm was moderated by self-control (*p* = 0.037). A similar pattern was observed whereby the association was stronger when self-control was low (1SD below the mean; OR = 3.72, 95% CI = 2.80, 4.95), in comparison to when self-control was high (1SD above the mean; OR = 2.60, 95% CI = 2.07, 3.26). There was no evidence for a moderating effect of either empathy (*p* = 0.932; 1SD below the mean: OR = 3.07, 95% CI = 2.36, 3.99; 1SD above the mean: OR = 3.03, 95% CI = 2.39, 3.84) or responsibility (*p* = 0.560; 1SD below the mean: OR = 3.17, 95% CI = 2.50, 4.03; 1SD above the mean: OR = 2.85, 95% CI = 2.14, 3.80). Evidence for the interactive effect of assertion remained when excluding cannabis (2-way *p* values: *p*_empathy_ = 0.990, *p*_assertion_ = 0.038, *p*_responsbility_ = 0.644, *p*_self-control_ = 0.218; Figure S1). Findings were consistent when examined across each period of young adulthood (all 3-way interaction *p* values > 0.05, Figure S2).

## Discussion

Findings from this study show shifts in the patterns of illicit substance use over time across young adulthood. Although cannabis remained the most used substance over time, there was also a transition in most used non-cannabis substances from hallucinogens to ecstasy and amphetamines. A majority of people who used substances also reported concurrent use by peers or partners. For social skills, responsibility was associated with lower levels of substance use and assertion with greater use. Greater levels of both assertion and self-control also buffered risk relationships between the number of illicit substances used and associated harm. Current findings support a potential role of intervening around social skills to minimise harms associated with substance use, in particular, interventions that promote aetiologically relevant social competencies, particularly assertion and self-control, that may protect against the adverse effects of illicit substance use. However, the complexity of associations attributed to assertion may suggest that it is important to determine which aspects are risky versus protective.

### Patterns of Substance Use in Young Adulthood

We identified several patterns in the use of illicit substances use across the young adulthood period. First, although the prevalence of any illicit substance use peaked at 27–28 years, the prevalence of non-cannabis illicit substance use, the number of substances used, and the experience of substance related harm peaked at age 23–24 years, which is consistent with other studies identifying similar peaks in illicit substance use around the mid-20 s (Chen & Jacobson, 2012). Second, patterns of illicit substance use varied by age. Whilst cannabis was the most used substance across all ages, there was a transition in the most used non-cannabis substances from hallucinogens at age 19–20 years to ecstasy and amphetamine use at ages 23–24 and 27–28 years.

Observed age related variations may contribute to understanding of the “gateway” sequence of substance use progression (Kandel, 1975; Yamaguchi & Kandel, 1984a, b), although more recent evidence suggests a more nuanced storey of drug progression (Degenhardt et al., 2010; Nkansah-Amankra & Minelli, 2016). For instance, it may also be that age-related changes in the patterns of substance use reflect sociocultural and secular influences on substance use choices such as changes in the availability of substance types (Australian Crime Commission, 2012, 2019) and social context (Arnett, 2005). In line with this, evidence from the current study suggested that the prevalence of peer use was lower towards the end of young adulthood. Nevertheless, some aspects of the development of illicit substance use have been commonly observed across decades and cultures, such as the “problem behaviour” construct whereby different forms of drug use and antisocial activities are behaviourally and socially clustered (Rowland et al., 2021). Further research would benefit from examining change in substance use behaviours, both individually and within close relationships, using trajectory-based modelling approaches, although the low prevalence of behaviours may cause model estimation difficulties.

Further, the majority of people who used substances at each young adulthood period also reported concurrent use by peers and/or partners. This finding is consistent with the overarching tenants of the social development model (Cambron et al., 2019; Catalano & Hawkins, 1996), which suggests that substance use behaviours often occur within social contexts, and whether the mechanism represents social selection (i.e., selecting peers with similar values and beliefs around substance use), social reinforcement (i.e., peers encouraging and supporting engagement in substance use), or potentially both; social connections are likely to be important in maintaining individual substance use behaviours. Interestingly, associations between illicit substance use and peer/partner use were notably stronger for peers than partners, which may suggest that during young adulthood prevention and intervention messaging might target social skills that help young people to with managing peer factors (e.g., peer pressure to engage in substance use).

### Social Skills and Individual Substance Use and Harm

Regarding social skills specifically, results suggested that greater levels of responsibility were associated with a reduction in any illicit substance use, supporting previous findings on social responsibility (Wray-Lake et al., 2012). More responsible adults may choose to avoid situations in which illicit substance use occurs or chooses to abstain in situations where illicit substances are available. In contrast, assertion was associated with higher levels of any illicit substance use. Results are consistent with findings from studies measuring non-substance-specific assertion, including elements closely related to social interactions (e.g., initiate conversations and express wishes clearly), which has been found to be associated with increased use (Goldberg-Lillehoj et al., 2005; Wills et al., 1989). Although assertive skills more specific to substance use behaviours (e.g., substance use refusal skills) have been associated with reduced substance use (Wills et al., 1989).

The strong relationship between the number of illicit substances used and the experience of harm was moderated by both assertion and self-control, such that the risk associations were weaker when levels of assertion and self-control were higher. In the current study, both the assertion and self-control scales encompass skills which are likely to help during situations involving decision making when under social pressure (e.g., assertion: “I express my wishes clearly, and give reasons for my actions and positions”; self-control: “I negotiate and compromise with people when we have disagreements”). These findings are supportive of the small, but closely comparable body of prior literature which also found greater self-control contributed to a weaker relationship between substance use and attributable problems (Simons et al., 2009; Wills et al., 2002, 2011). Further, given the current measurements, findings lend additional support to the importance of behavioural control mechanisms identified previously (Wills et al., 2011). Together, it is possible that a reduction in the strength of association between increased use and harm may reflect an increased ability to appropriately navigate social situations and in turn make better decisions. However, it is important to note that these associations may be structured by even ‘deeper’ aetiological processes such as those related to underlying personality traits (Caspi et al., 2014). This is an area that warrants further research in the future.

### Limitations

In mature cohort studies, there are important sources of selection, measurement, and confounding bias that require consideration (Sterne et al., 2016). Although multiple imputation was used to minimise missing data bias in inferential analyses, it is likely that there was some selective attrition of the most vulnerable individuals, such as those with high levels of substance use. Whether or not social skills protect against the relationship between use and harm in more vulnerable users requires further exploration. Further, descriptive statistics, which are not based on imputed data, may be further impacted by selection bias, although differences in the prevalence of key sociodemographic baseline characteristics appear relatively minor across each young adult wave. It is also important to note that the young adult data span from the early 2000s to 2010, and contemporary patterns of use may vary. Additionally, as all measures are self-report, shared method variance, social desirability bias, and perception bias may be issues. Furthermore, there was some misalignment in item wording about the timing of illicit substance use (i.e., past month use) compared to illicit harm (i.e., past 12 months), meaning that illicit harm could be related to other aspects of illicit substance use not captured in the current data. Additionally, whilst the current study could examine the number of substances used in the past month, the use of multiple substances on a single occasion was not able to be determined. Finally, whilst we included a range of potential confounding factors, future work is needed to further explore causal pathways.

### Conclusions

Using multi-wave longitudinal data across young adulthood, the current study found that although the frequency of any illicit substance use peaked at 27–28 years, the number of used substances and substance related harm peaked at age 23–24 years. Cannabis was the most used substance, and there was a transition in the most used non-cannabis substances from hallucinogens in the early 20 s to ecstasy and amphetamines later in the 20 s. Further, in regard to social factors, peer and partner use, lower responsibility, and higher assertion were associated with increased illicit substance use. Importantly, assertion and self-control had a protective role in buffering the risk relationships between the number of illicit substances used and harm. Taken together, findings from the current study suggest that interventions which aim to strengthen targeted social skills prior to, or during young adulthood, may play a role in protecting against illicit substance use and associated harm in young people.

## Supplementary Information

Below is the link to the electronic supplementary material.

Supplementary file1 (XLSX 35 KB)

Supplementary file2 (DOCX 1036 KB)

## Data Availability

While study protocols do not permit potentially re-identifiable participant data to be made publicly available, we welcome collaboration with the ATP research team subject to appropriate permissions and ethical approval. Enquiries about collaboration are possible through our institutional data access protocol: https://lifecourse.melbournechildrens.com/data-access/.
