# Supplementary material for: Illicit Substance Use and Harm in Young Adulthood: the Role of Substance Use in Close Relationships and Individual Social Skills
Source: Int J Ment Health Addict. 2023 Oct 27;23(2):1503–17. doi: 10.1007/s11469-023-01181-0 (PMC12041169; doi:10.1007/s11469-023-01181-0)
Supplement: Supplementary file 2 — Supplementary file2 (DOCX 1036 KB) [file 11469_2023_1181_MOESM2_ESM.docx]

Supplementary Materials

| Table S2. Associations between illicit substance use (excluding cannabis) and both social skills and social use indicators (N=1,404) | | | | |
| --- | --- | --- | --- | --- |
|  | Model 1 | | Model 2 | |
|  | OR | 95% CI | OR | 95% CI |
| Social Skills |  |  |  |  |
| Empathy (z-score) | 0.99 | (0.87, 1.12) | 0.93 | (0.80, 1.09) |
| Assertion (z-score) | 1.21 | (1.07, 1.38) | 1.32 | (1.13, 1.55) |
| Responsibility (z-score) | 0.79 | (0.70, 0.88) | 0.81 | (0.70, 0.92) |
| Self-control (z-score) | 0.98 | (0.87, 1.10) | 1.00 | (0.86, 1.16) |
| Social use |  |  |  |  |
| Peer use | 5.71 | (4.16, 7.85) | 4.46 | (3.18, 6.24) |
| Partner use | 2.80 | (2.08, 3.75) | 2.53 | (1.86, 3.46) |
| Note: Model 1 = adjusted for measurement time point only; Model 2 = additionally adjusted for social skills, peer/partner substance use, and potential confounding factors. | | | | |

| Table S3. Associations between any illicit substance use and both social skills and social use indicators by young adult age group (N=1,404) | | | | | | | |
| --- | --- | --- | --- | --- | --- | --- | --- |
|  | 19-20 years | | 23-24 years | | 27-28 years | | Interaction |
|  | OR | 95% CI | OR | 95% CI | OR | 95% CI | p-value |
| Including Cannabis |  |  |  |  |  |  |  |
| Empathy (z-score) | 1.01 | (0.86, 1.20) | 0.85 | (0.71, 1.02) | 1.08 | (0.90, 1.31) | 0.079 |
| Assertion (z-score) | 1.24 | (1.05, 1.45) | 1.17 | (0.98, 1.40) | 1.33 | (1.12, 1.59) | 0.529 |
| Responsibility (z-score) | 0.76 | (0.66, 0.88) | 0.72 | (0.60, 0.87) | 0.88 | (0.75, 1.04) | 0.156 |
| Self-control (z-score) | 1.10 | (0.95, 1.29) | 0.95 | (0.80, 1.13) | 1.09 | (0.92, 1.29) | 0.307 |
| Social use |  |  |  |  |  |  |  |
| Peer use | 7.41 | (4.75, 11.54) | 4.39 | (2.86, 6.75) | 4.35 | (3.11, 6.09) | 0.162 |
| Partner use | 3.76 | (2.54, 5.56) | 4.25 | (2.73, 6.63) | 2.90 | (1.87, 4.49) | 0.418 |
| Excluding Cannabis |  |  |  |  |  |  |  |
| Empathy (z-score) | 1.00 | (0.79, 1.27) | 0.81 | (0.66, 0.99) | 1.08 | (0.86, 1.37) | 0.052 |
| Assertion (z-score) | 1.33 | (1.04, 1.70) | 1.23 | (1.01, 1.50) | 1.47 | (1.16, 1.87) | 0.367 |
| Responsibility (z-score) | 0.73 | (0.59, 0.90) | 0.76 | (0.62, 0.92) | 0.96 | (0.77, 1.19) | 0.103 |
| Self-control (z-score) | 0.97 | (0.76, 1.25) | 0.96 | (0.80, 1.16) | 1.06 | (0.83, 1.35) | 0.764 |
| Social use |  |  |  |  |  |  |  |
| Peer use | 6.54 | (3.06, 13.99) | 4.86 | (2.85, 8.29) | 3.54 | (2.13, 5.88) | 0.379 |
| Partner use | 1.91 | (1.09, 3.36) | 3.46 | (2.22, 5.40) | 2.03 | (1.19, 3.49) | 0.120 |
| Note: Models adjusted for social skills, peer/partner substance use, and potential confounding factors | | | | | | | |

| Table S4. Associations between illicit substance use harm and the number of illicit substances used by young adult age group (N=1,404) | | | | | | | |
| --- | --- | --- | --- | --- | --- | --- | --- |
|  | 19-20 years | | 23-24 years | | 27-28 years | | Interaction |
|  | OR | 95% CI | OR | 95% CI | OR | 95% CI | p-value |
| Including Cannabis |  |  |  |  |  |  |  |
| Number of illicit substances | 3.30 | (2.35, 4.65) | 2.94 | (2.31, 3.76) | 3.12 | (2.30, 4.24) | 0.838 |
| Excluding Cannabis |  |  |  |  |  |  |  |
| Number of illicit substances | 5.24 | (2.83, 9.70) | 4.14 | (3.01, 5.69) | 3.78 | (2.59, 5.53) | 0.665 |
| Note: Models adjusted for social skills, peer/partner substance use, and potential confounding factors | | | | | | | |

| 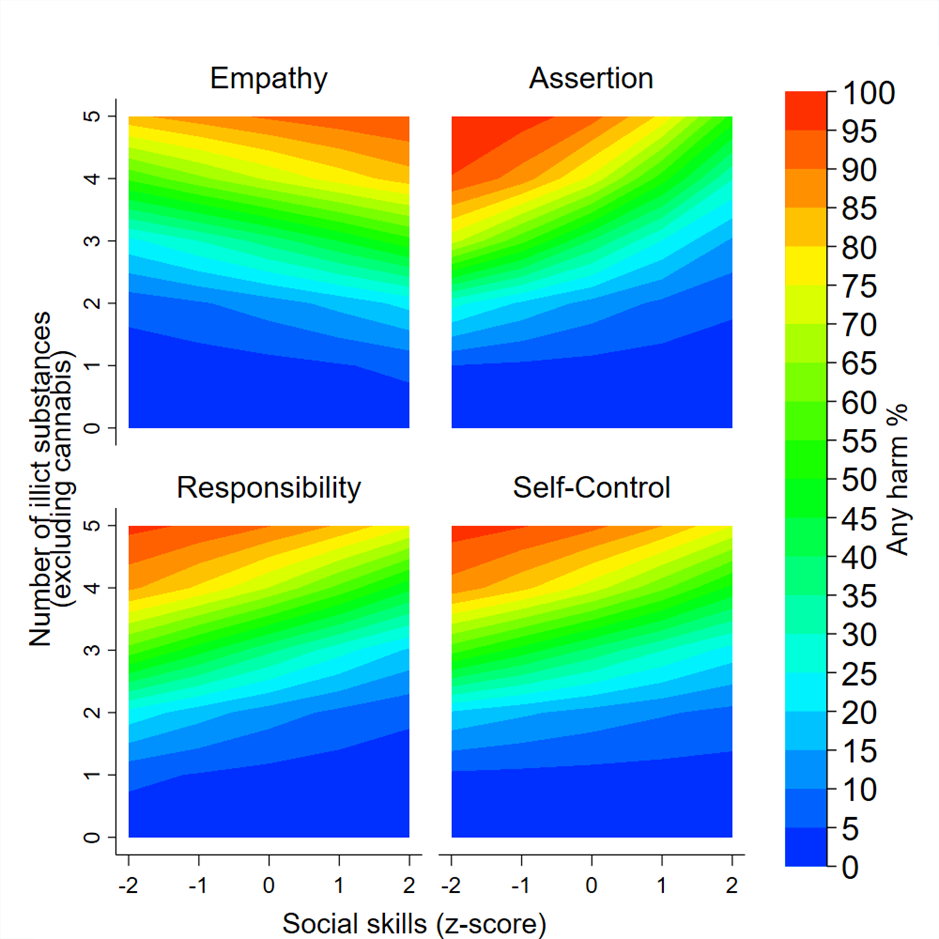 |
| --- |
| Figure S1. Percentage of participants experiencing harm in the interactive effect of the number of illicit substances and social skills (excluding cannabis).  Note: Number of potential illicit substances excluding cannabis ranges from 0 to 5. |

| 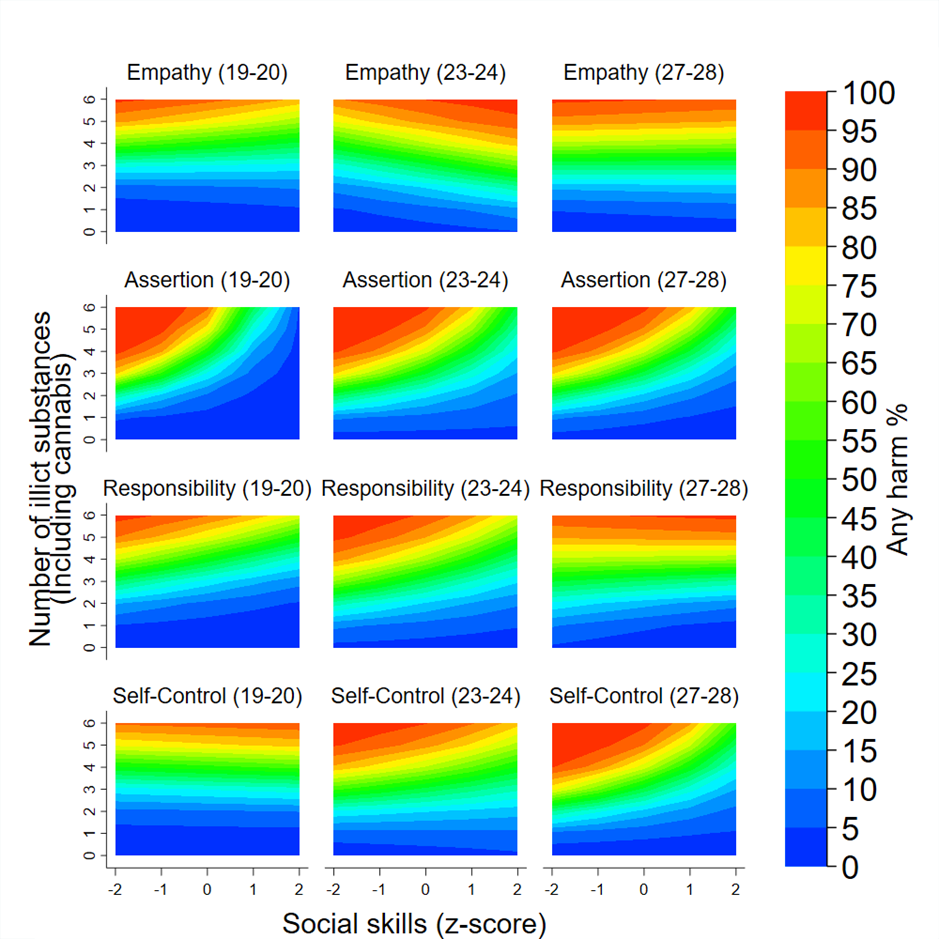 | 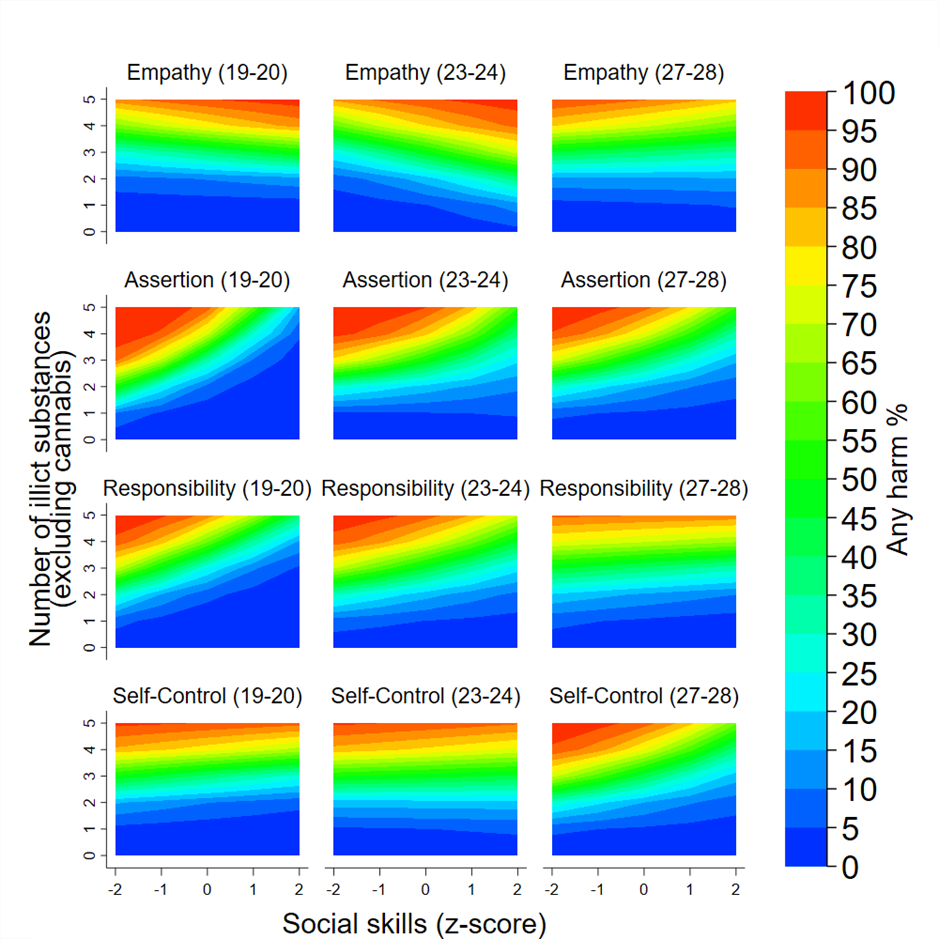 |
| --- | --- |
| Figure S2. Percentage of participants experiencing harm in the interactive effect of the number of illicit substance and social skills by young adult age group  Note: Number of potential illicit substances including cannabis ranges from 0 to 6 and excluding cannabis ranges from 0 to 5. | |
